# Efficacy of Teduglutide for Parenteral Support Reduction in Patients with Short Bowel Syndrome: A Systematic Review and Meta-Analysis

**DOI:** 10.3390/nu14040796

**Published:** 2022-02-14

**Authors:** Fabio Bioletto, Chiara D’Eusebio, Fabio Dario Merlo, Umberto Aimasso, Marta Ossola, Marianna Pellegrini, Valentina Ponzo, Alessia Chiarotto, Antonella De Francesco, Ezio Ghigo, Simona Bo

**Affiliations:** 1 Department of Medical Sciences, University of Turin, 10126 Turin, Italy; fabio.bioletto@unito.it (F.B.); chiara.deusi@gmail.com (C.D.); mariannapellegrini87@gmail.com (M.P.); valentina.ponzo@unito.it (V.P.); ezio.ghigo@unito.it (E.G.); 2 Dietetic Unit, Città della Salute e della Scienza Hospital, 10126 Turin, Italy; fdmerlo@gmail.com (F.D.M.); uaimasso@cittadellasalute.to.it (U.A.); mossola@cittadellasalute.to.it (M.O.); alessia.chiarotto@unito.it (A.C.); defrancesco54anto@gmail.com (A.D.F.)

**Keywords:** teduglutide, short bowel syndrome, chronic intestinal failure, parenteral nutrition, parenteral support

## Abstract

Teduglutide has been described as an effective treatment for parenteral support (PS) reduction in patients with short bowel syndrome (SBS). However, a quantitative summary of the available evidence is still lacking. PubMed/Medline, EMBASE, Cochrane library, OVID, and CINAHL databases were systematically searched up to July 2021 for studies reporting the rate of response (defined as a ≥20% reduction in PS) to teduglutide among PS-dependent adult patients. The rate of weaning (defined as the achievement of PS independence) was also evaluated as a secondary end-point. Ten studies were finally considered in the meta-analysis. Pooled data show a response rate of 64% at 6 months, 77% at 1 year and, 82% at ≥2 years; on the other hand, the weaning rate could be estimated as 11% at 6 months, 17% at 1 year, and 21% at ≥2 years. The presence of colon in continuity reduced the response rate (−17%, 95%CI: (−31%, −3%)), but was associated with a higher weaning rate (+16%, 95%CI: (+6%, +25%)). SBS etiology, on the contrary, was not found to be a significant predictor of these outcomes, although a nonsignificant trend towards both higher response rates (+9%, 95%CI: (−8%, +27%)) and higher weaning rates (+7%, 95%CI: (−14%, +28%)) could be observed in patients with Crohn’s disease. This was the first meta-analysis that specifically assessed the efficacy of teduglutide in adult patients with SBS. Our results provide pooled estimates of response and weaning rates over time and identify intestinal anatomy as a significant predictor of these outcomes.

## 1. Introduction

Short bowel syndrome (SBS), i.e., the condition following surgical removal of a portion of the small bowel until the intestinal length is shorter than 200 cm, could be caused by different underlying diseases such as mesenteric ischemia, Crohn’s disease, radiation enteritis, and surgical complications, among others [1,2,3,4,5]. SBS is the most frequent etiology of chronic intestinal failure (CIF) [1,2,3,4,5]. CIF is defined as the reduction in gut function below the minimum necessary for the absorption of macronutrients and/or water and electrolytes, such that intravenous supplementation is required to maintain health [1,2,3]; until 1970 [6], CIF was a condition incompatible with life; this is still true in some countries nowadays. Parenteral support (PS), i.e., parenteral nutrition (PN) and/or intravenous fluid administration, revolutionized the prognosis of patients with CIF [7,8]. Nevertheless, patients with CIF needing chronic PS are still burdened by many complications, such as liver, kidney, and bone disease, acid–base dysregulations, sepsis, catheter thrombosis, and poor quality of life (QoL) [9,10,11,12,13,14,15]. Those complications, whose probability is higher as time on PS increases, generate high healthcare costs, which have to be added to those produced by PS itself [16,17].

Teduglutide, an analog of the intestinotrophic hormone glucagon-like-peptide 2 (GLP-2) secreted by the intestinal L-cells, is an effective therapeutic option for patients with SBS-related CIF [18,19,20]. GLP-2 receptor activation determines, through multiple pathways and mediators, a hypertrophy effect on bowel mucosa, increasing villus height and crypt depth [21,22]; moreover, it also inhibits gastric acid secretions [23], delays gastric emptying [24], stimulates intestinal blood flow [25], and opposes inflammatory insults [26]. Teduglutide treatment, thus, leads to an overall increase in the intestinal absorption capacity, reducing PS needs. Its efficacy in PS reduction was first demonstrated in adult patients [27,28] and, more recently, it has shown effectiveness in children [29,30], with similar results. Weaning off PS, or even just a reduction in volumes and days of weekly infusions, may reduce PS-related healthcare costs and increase the QoL of SBS patients [31]. Teduglutide is, to date, an expensive treatment, but cost effectiveness analyses affirmed that its use could be cost saving in selected subgroups of patients [32]. Previous studies have demonstrated that the teduglutide response in patients with SBS is highly heterogeneous, most likely due to differences in SBS etiology, intestinal anatomy, and PS volume requirements [33,34,35]. The same holds for the achievement of PS independence, but factors explaining this heterogeneity are less clear, mostly due to the limited number of available patients [36,37]. A better understanding of the predictors of teduglutide efficacy may improve patient management and profiling, as well as optimize the use of healthcare resources [34,35].

The aim of the present systematic review and meta-analysis was to evaluate the efficacy of teduglutide for PS reduction in adult patients with SBS; moreover, its efficacy for PS weaning was assessed. The possible roles of intestinal anatomy and SBS etiology as predictors of these outcomes were also analyzed.

## 2. Materials and Methods

### 2.1. Search Strategy and Study Selection

This study was conducted according to the Preferred Reporting Items for Systematic Reviews and Meta-Analysis (PRISMA) guidelines [38]. The process of literature search and study selection was carried out by three independent reviewers (C.D.E., F.B., V.P.); all disparities were resolved through consensus.

The following electronic databases were queried until 1 July 2021: PubMed/Medline, EMBASE, Cochrane library, OVID, and CINAHL. The search strategy was performed using a combination of relevant database-specific search terms to identify pertinent studies on the efficacy of teduglutide for parenteral support reduction in adult patients with SBS. Both medical subject headings (MeSH) and free-text search terms were employed. The term “teduglutide” was combined with other key words such as short bowel, short gut, intestinal failure, intestinal insufficiency, intestinal deficiency, intestinal growth, intestinal adaptation, parenteral nutrition, parenteral support, complications, cancer, and dysplasia. The full search strategy is presented in Appendix A. No filters were applied for study design, language, or publication date. To expand the search, references of the retrieved articles were also screened for additional studies.

After duplicate removal, all studies found with the aforementioned search were evaluated for inclusion in the meta-analysis, first by title/abstract screening and then by full-text review. We excluded studies from our analysis according to the following exclusion criteria: (a) evaluation of teduglutide treatment efficacy in pediatric patients; (b) evaluation of teduglutide treatment efficacy only at <24 weeks; (c) evaluation of teduglutide treatment efficacy at undefined or poorly defined time points; (d) evaluation of <10 PS-dependent patients treated with teduglutide; and (e) unavailability of the primary outcome of interest, as defined in the following subsection. Post hoc analyses of included studies were assessed for relevance and employed for data extraction if they contained additional information for the assessment of the outcomes of interest. In case of patient overlap between studies to be pooled in the same analysis, the one with the largest sample size was considered.

### 2.2. Outcomes

The primary outcome of interest was the proportion of patients that achieved PS reduction, defined as a ≥20% reduction in PS volume with respect to baseline; these data will be referred to as “response rate” throughout the rest of the manuscript. The secondary outcome was the proportion of patients that achieved complete PS independence; these data will be referred to as “weaning rate” throughout the rest of the manuscript. Comparisons of response and weaning rates according to intestinal anatomy and SBS etiology were performed based on crude rate differences.

### 2.3. Data Extraction

Three authors (C.D.E., F.B., V.P.) independently examined and extracted data from papers which met the inclusion criteria using prespecified data extraction templates. For each eligible study, the following information was collected: (a) first author and publication year; (b) study design; (c) number of subjects; (d) patients’ characteristics in terms of demographic data and SBS-related data; (e) time points at which the efficacy of teduglutide was assessed; (f) observed proportion of patients that achieved a ≥20% reduction in PS volume at each considered time-point (response rate); (g) observed proportion of patients that achieved a complete independence from PS at each considered time-point (weaning rate); (h) stratification of response rate and weaning rate according to intestinal anatomy, if available; and (i) stratification of response rate and weaning rate according to SBS etiology, if available.

### 2.4. Risk of Bias Assessment

The risk of bias was independently assessed for each included study by two authors (F.B., S.B.) using the RoB 2 (Risk of Bias) tool for randomized controlled trials [39] and the ROBINS-I (Risk Of Bias In Non-randomized Studies of Intervention) tool for observational studies [40].

The first tool evaluated five domains, which addressed (a) bias arising from the randomization process, (b) bias due to deviations from intended interventions, (c) bias due to missing outcome data, (d) bias in measurement of the outcome, and (e) bias in selection of the reported result. An additional evaluation of overall risk of bias was also carried out as a summary measure. The options for a domain-level risk-of-bias judgement were ‘Low’, ‘Some concerns’ or ‘High’. The second tool evaluated seven domains, which addressed (a) bias due to confounding, (b) bias in selection of participants for the study, (c) bias in measurement classification of interventions, (d) bias due to deviations from intended interventions, (e) bias due to missing data, (f) bias in measurement of outcomes, (g) bias in selection of the reported result. An additional evaluation of overall risk of bias was also carried out as a summary measure. The options for a domain-level risk-of-bias judgement were ‘Low’, ‘Moderate’, ‘Serious’ or ‘Critical’, with an additional option of ‘Unknown’ if sufficient information for judgement was lacking.

### 2.5. Statistical Analysis

Continuous variables and categorical variables were reported as numbers and percentages, respectively. The proportion of patients that achieved the outcomes of interest was assessed as an absolute rate and compared between groups as a rate difference. A random-effect model with inverse-variance weighting was adopted for statistical pooling of the retrieved data [41]. Higgins I^2^ statistics and Cochran Q-test were used to assess heterogeneity between studies [42]. Publication bias was quantitatively assessed by Begg’s test. Statistical analysis was performed using R 4.0.3 (R Core Team, R Foundation for Statistical Computing, Vienna, Austria) and STATA 17 (StataCorp, College Station, TX, USA).

## 3. Results

### 3.1. Search Results

**Figure 1:**
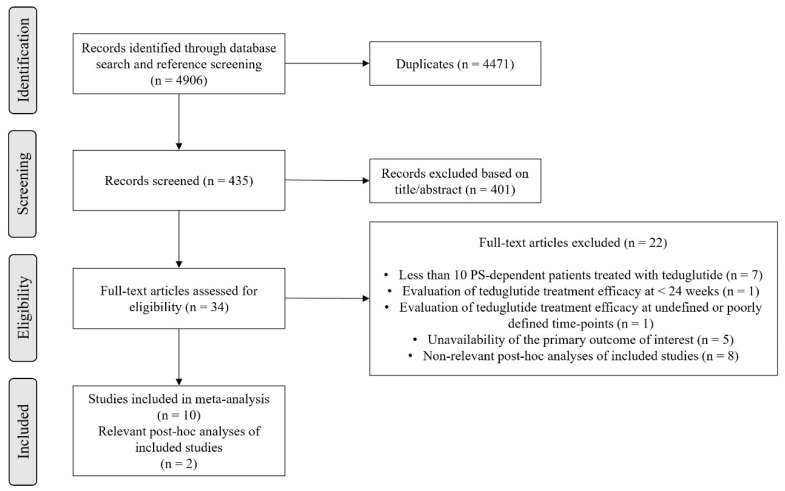
Flow-chart of study inclusion. Abbreviations: PS, parenteral support.

A total of 4906 records were identified in the initial literature search (accounting for possible duplicates within each electronic database if a record was obtained in multiple search strings). Removal of duplicates led to an overall pool of 435 studies. An accurate title or abstract revision was sufficient to exclude 401 articles as not pertinent or not fulfilling our prespecified inclusion or exclusion criteria. The remaining 34 studies were assessed in full-text for eligibility; 10 of them finally met all criteria for being included in the final analysis [27,28,43,44,45,46,47,48,49,50]; 2 additional post hoc analyses were employed for data extraction [35,37], as they contained additional and relevant information for the assessment of the outcomes of 3 among the 10 pertinent papers [28,45,49]. The process of study selection is summarized in Figure 1.

### 3.2. Characteristics of the Included Studies

Table 1 summarizes the basic study characteristics. Two studies had a randomized, placebo-controlled, double-blinded design [27,28]; the remaining eight studies were characterized by an observational design [43,44,45,46,47,48,49,50]. Among these, one was actually designed as a randomized controlled trial (RCT) [45], but the randomization and the blindness of treatment assignment was performed between different teduglutide dosing schedules rather than between the teduglutide and placebo; therefore, to the scope of our analyses, this study falls in the group of “observational” ones, since both the investigators and the patients were aware that a treatment with the drug was actually in place.

The teduglutide dose was 0.05 mg/kg/day in most studies, with the only exception of two studies, in which the 0.1 mg/kg/day dose was also used [27,45]. As for the considered time points at which the efficacy of the teduglutide treatment was assessed, both the available RCTs evaluated their end-points after 6 months of treatment [27,28]; an additional four observational studies provided efficacy data at this time-point [43,46,48,50]. Five studies, all observational, evaluated teduglutide efficacy after 1 year of treatment [44,45,46,47,48]; three studies provided data about teduglutide efficacy after ≥2 years of treatment [46,47,49] (Table 2).

### 3.3. Efficacy of Teduglutide in Achieving PS Reduction

**Figure 2:**
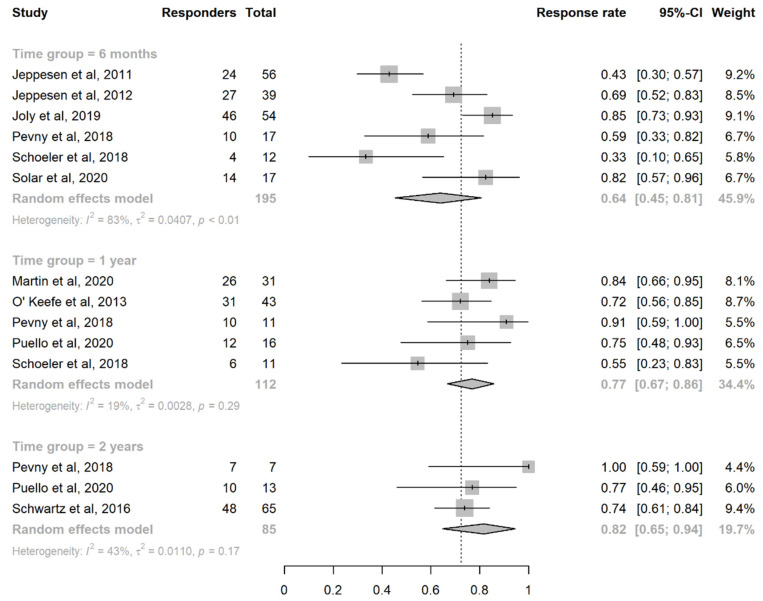
Forest plot for the estimation of the response rate at 6 months, 1 year, and ≥2 years. Abbreviations: CI, confidence interval.

Considering the two available placebo-controlled trials [27,28], the response rate was significantly greater in the teduglutide than in the placebo group (+36%, 95%CI: (+22%, +50%)) (Figure S1). When pooling the efficacy results of the treatment arms of these trials [27,28] with those of the observational studies that evaluated the primary outcome of interest at 6 months [43,46,48,50], the pooled estimate of the response rate was 64% (95%CI: (45%, 81%)) (Figure 2), without a significant difference between RCTs and observational studies (*p* = 0.50) (Figure S2). At 1 year [44,45,46,47,48], the pooled estimate of the response rate was 77% (95%CI: (67%, 86%)) (Figure 2). At ≥2 years [46,47,49], the pooled estimate of the response rate was 82% (95%CI: (65–94%)) (Figure 2). All these analyses were repeated after excluding the patients treated with a dosing schedule of 0.1 mg/kg/day, with the overall results being substantially confirmed (Figures S3–S5).

**Figure 3:**
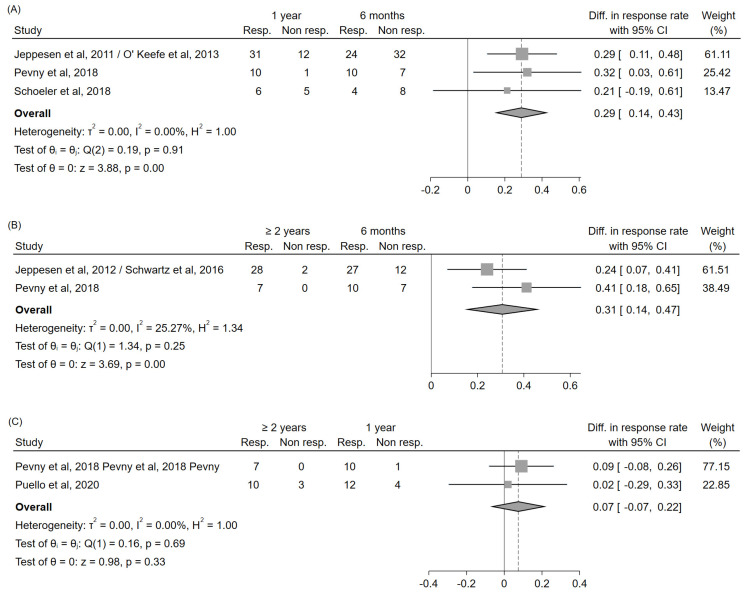
Forest plot for the estimation of the differences in the response rates over time, evaluated in studies reporting these data in the same patient cohort at different time points (1 year vs. 6 months (**A**); ≥2 years vs. 6 months (**B**); ≥2 years vs. 1 year (**C**)). Abbreviations: CI, confidence interval; Diff., difference; Resp., responders; Non resp., nonresponders; vs., versus.

In order to specifically explore possible differences in teduglutide efficacy over time, studies reporting the response rate in the same patient cohort at different time points were selected [27,28,45,46,47,48,49], and differences in the response rate over time were evaluated. A significant increase in response rate could be noted between 6 months and 1 year (+29%, 95%CI: (+14%, +43%)), and between 6 months and ≥2 years (+31%, 95%CI: (+14%, +47%)); on the contrary, no significant differences emerged between 1 year and ≥2 years (+7%, 95%CI: (–7%, +22%)), even if a trend towards increase could still be noted (Figure 3).

### 3.4. Efficacy of Teduglutide in Achieving PS Independence

**Figure 4:**
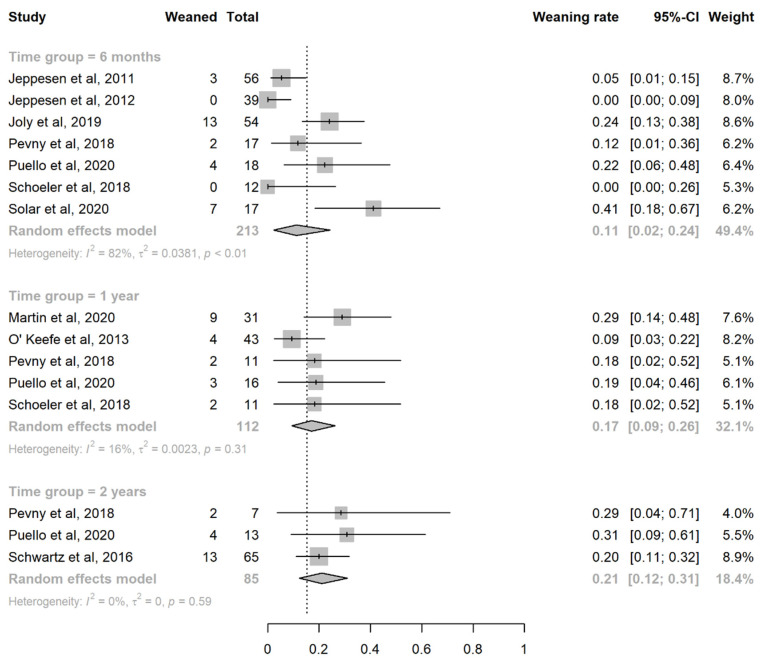
Forest plot for the estimation of the weaning rate at 6 months, 1 year, and ≥2 years. Abbreviations: CI, confidence interval.

All included studies also evaluated, at the same time points as the primary outcome measure, the proportion of teduglutide-treated patients that could achieve PS independence (weaning rate). The pooled estimates for this outcome were 11% (95%CI: (2%, 24%)) at 6 months [27,28,43,46,47,48,50], 17% (95%CI: (9%, 26%)) at 1 year [44,45,46,47,48], and 21% (95%CI: (12%, 31%)) at ≥2 years [46,47,49] (Figure 4). Overall, these results were confirmed even after excluding the patients treated with a dosing schedule of 0.1 mg/kg/day (Figure S6).

**Figure 5:**
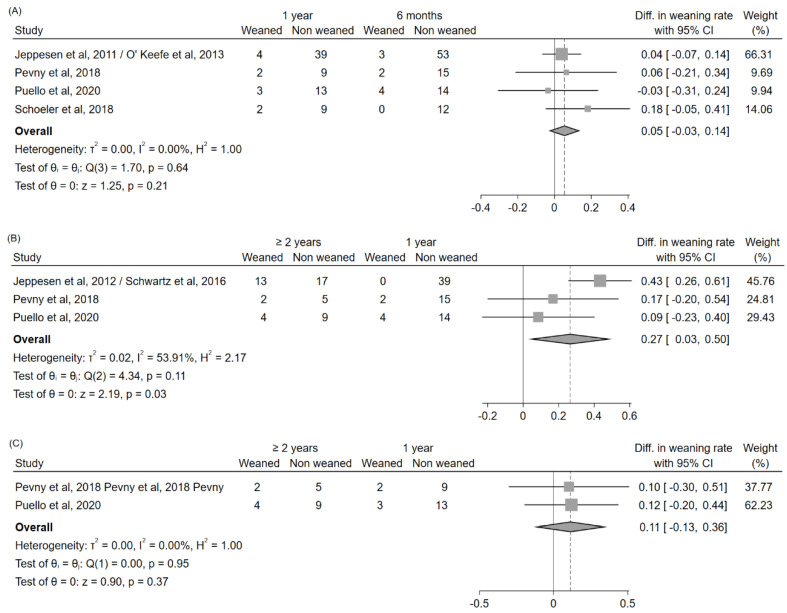
Forest plot for the estimation of the differences in the weaning rate over time, evaluated in studies reporting these data in the same patient cohort at different time points (1 year vs. 6 months (**A**); ≥2 years vs. 6 months (**B**); ≥2 years vs. 1 year (**C**)). Abbreviations: CI, confidence interval; Diff., difference; vs., versus.

In order to specifically explore possible differences in teduglutide efficacy over time, studies reporting the weaning rate in the same cohort at different time points were selected [27,28,45,46,47,48,49], and differences over time were evaluated. A significant increase in weaning rate could be noted between 6 months and ≥2 years (+27%, 95%CI: (+3%, +50%)), mostly driven by the patient cohort analyzed in the studies by Jeppesen et al. [28] and Schwartz et al. [49]; on the contrary, no significant differences emerged between 6 months and 1 year (+5%, 95%CI: (–3%, +14%)) or between 1 year and ≥2 years (+11%, 95%CI: (–13%, +36%)), even if a trend towards increase could still be noted (Figure 5).

### 3.5. Relationship between Teduglutide Efficacy and Intestinal Anatomy

**Figure 6:**
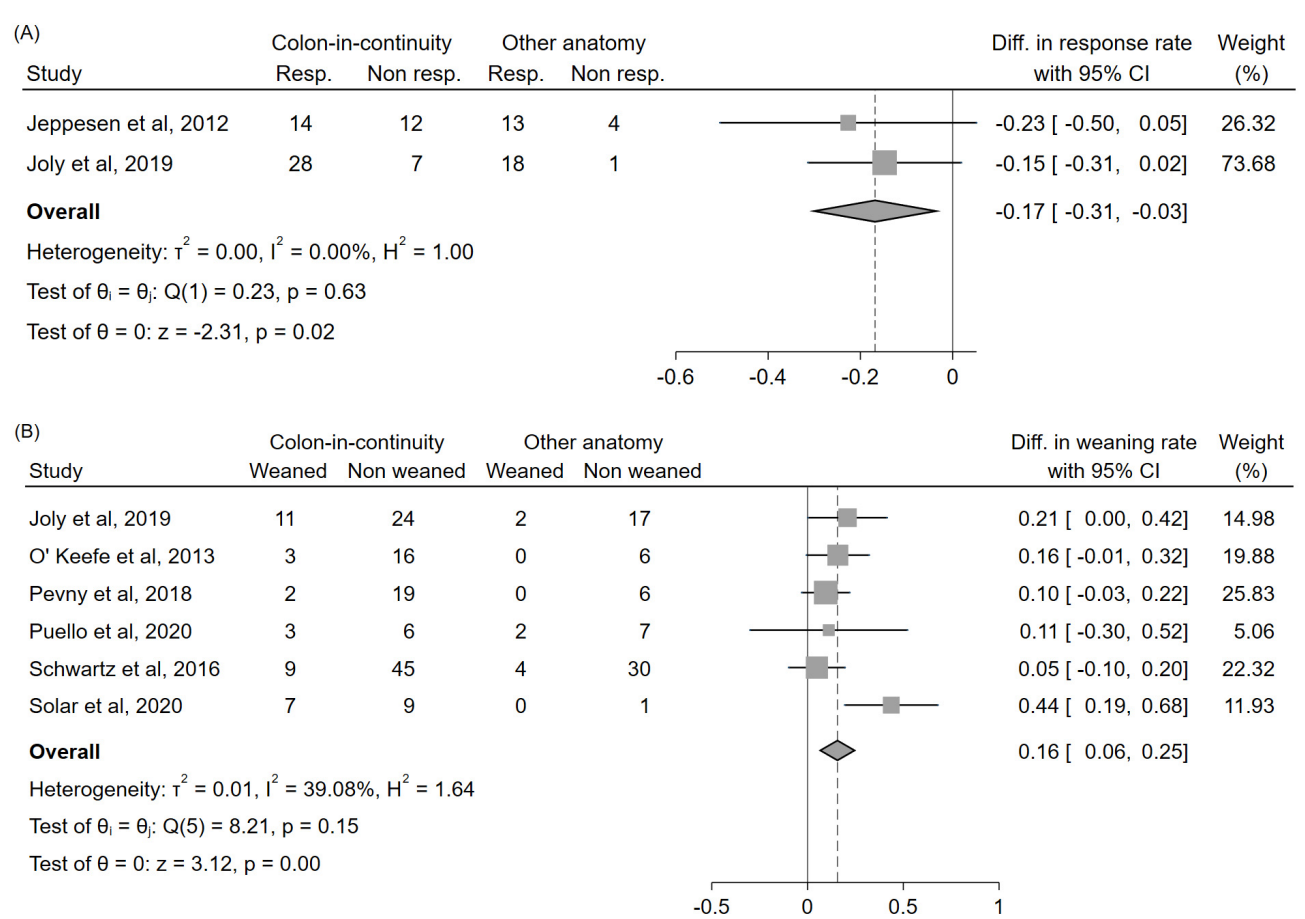
Forest plot comparing the response rate (**A**) and the weaning rate (**B**) according to the presence or the absence of colon in continuity. Reported results did not account for dropouts due to insufficient data. In the study by O’Keefe et al., only the data concerning patients treated with the 0.05 mg/kg/day dosing schedule could be extracted. Abbreviations: CI, confidence interval; Diff., difference; Resp., responders; Non resp., non responders.

Three studies [28,43,44] reported sufficient data to compare the response rate among patients with or without colon in continuity; two of them evaluated this outcome at 6 months [28,43], and one at 1 year [44]; the latter [44], however, had to be excluded from the analysis due to partial patient overlap with one of the previous two [43]. The response rate was significantly lower (–17%, 95%CI: (–31%, –3%)) among patients with colon in continuity (Figure 6A). Six studies [43,45,46,47,49,50] reported sufficient data to compare the weaning rate among those with or without colon in continuity; one of them evaluated this outcome at 6 months [43], one at 1 year [45], one at ≥2 years [49], and three at the last available follow-up [46,47,50]. The weaning rate was significantly higher (+16%, 95%CI: (+6%, +25%)) among those with colon in continuity (Figure 6B). No further analyses could be conducted to differentiate the outcomes according to a finer classification of intestinal anatomy due to the paucity of available data; in particular, no sufficient data were available to compare these outcomes between patients with different types of stomas.

### 3.6. Relationship between Teduglutide Efficacy and SBS Etiology

**Figure 7:**
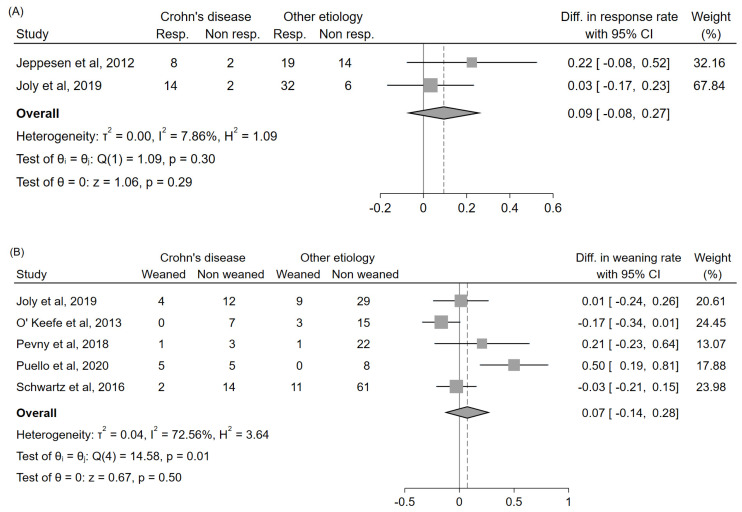
Forest plot comparing the response rate (**A**) and the weaning rate (**B**) according to SBS etiology. Reported results do not account for dropouts due to insufficient data. In the study by O’Keefe et al., only the data concerning patients treated with the 0.05 mg/kg/day dosing schedule could be extracted. Abbreviations: CI, confidence interval; Diff., difference; Resp., responders; Non resp., nonresponders.

Three studies [28,43,44] reported sufficient data to compare the response rate among patients with SBS due to Crohn’s disease or with SBS due to a different etiology; two of them evaluated this outcome at 6 months [28,43], and one at 1 year [44]; the latter [44], however, had to be excluded from the analysis due to partial patient overlap with one of the previous two [43]. The response rate did not significantly differ between the two groups, although a nonsignificant trend toward a slightly better outcome among patients with SBS due to Crohn’s disease could be observed (+9%, 95%CI: (−8%, +27%)) (Figure 7A). Five studies [43,45,46,47,49] reported sufficient data to compare the weaning rate among patients with SBS due to Crohn’s disease or with SBS due to a different etiology; one of them evaluated this outcome at 6 months [43], one at 1 year [45], one at ≥2 years [49], and two at the last available follow-up [46,47]. The weaning rate did not significantly differ between the two groups, although a nonsignificant trend toward a slightly better outcome among patients with SBS due to Crohn’s disease could be observed (+7%, 95%CI: (−14%, +28%)) (Figure 7B). No further analyses could be conducted to differentiate the outcomes according to a finer classification of SBS etiology due to the paucity of available data.

### 3.7. Quality Assessment and Publication Bias

The results of the quality assessment of the studies are reported in Tables S1 and S2. Altogether, the risk of bias was moderate-to-low in all studies, with the most relevant concerns being related to possible confounding and selection bias in some of the included observational studies. No significant publication bias was found with Begg’s test with respect to the primary outcome measure at any of the considered time points (*p* = 0.70 for the studies at 6 months, *p* = 0.80 for the studies at 1 year, *p* = 0.12 for the studies at ≥2 years).

## 4. Discussion

### 4.1. Overall Efficacy

The results of this systematic review and meta-analysis confirm the efficacy of teduglutide for parenteral support reduction in adult patients with SBS. As a primary outcome measure, we evaluated the proportion of patients achieving a ≥20% reduction in PS volume during teduglutide treatment (“response rate”); this choice was consistent with the available literature, in which this cut-off had been unanimously considered as the one of choice for the distinction between responders and non-responders. As a secondary outcome measure, we evaluated the proportion of patients achieving PS independence (“weaning rate”).

The results of our analyses show that the response rate to teduglutide treatment could be estimated as 64% at 6 months, 77% at 1 year, and 82% at ≥2 years; on the other hand, the weaning rate could be estimated as 11% at 6 months, 17% at 1 year, and 21% at ≥2 years. When specifically addressing the question of the course of the response to teduglutide over time, the response rate showed a significant increase between 6 months and 1 year and was at least maintained afterwards. Data about the weaning rate were less clear, even if a trend towards an increase in efficacy over time could still be noted. This result is in line with the notion that, as already supposed by other authors [35,45,49], the benefit of teduglutide treatment for PS volume reduction might not only be sustained, but likely increases over time after treatment initiation.

### 4.2. Efficacy by Intestinal Anatomy

Our analysis shows that the efficacy of teduglutide was significantly influenced by intestinal anatomy; the presence of colon in continuity reduced the response rate by −14%, but, conversely, was associated with an increase in weaning rate of +16%.

With respect to the response rate, our findings reinforce those already obtained by previous studies [33,34,35], in which lower PS volume reductions could be observed in patients with colon in continuity compared to those with jejunostomy/ileostomy. Phenotypically, patients with jejunostomy/ileostomy are frequently characterized by accelerated gastric emptying, poor adaptation following resection, and higher PS volume needs [33,51,52]. In these patients, a reduced endogenous GLP-2 secretion was reported, as a consequence of the resection of the terminal ileum and the colon, where the L-cells are predominantly located [33,53,54]; on the contrary, in patients with colon in continuity, the endogenous GLP-2 secretion is essentially preserved [33,53,54]. Therefore, patients experiencing the most pronounced benefits from the administration of an exogenous GLP-2 analog would most probably be those without colon in continuity.

Regarding the weaning rate, irrespectively to teduglutide treatment, the presence of colon in continuity is known to be associated with lower PS volume needs and a higher probability of progressive spontaneous adaptation with possible weaning from PS after an intestinal resection [2,55,56,57]. In general, patients with colon in continuity may have a greater innate potential for adaptation because of the colon’s role in fluid absorption and energy conservation [2,33]. Thus, a similar result could also be expected among teduglutide-treated patients. However, even though some authors have already pointed out a possible tendency towards a higher weaning rate among teduglutide-treated patients with colon in continuity compared to those with other intestinal anatomies [37,43,49], none of the published studies were able to reach a statistical significance on this outcome in univariate analysis, probably due to the low number of included patients. Therefore, our study was the first to actually prove a statistically significant difference in weaning rates between patients with and without colon in continuity.

In summary, the available evidence suggests that the presence/absence of colon in continuity predicts in a different and opposite way the achievement of response and/or weaning after the initiation of teduglutide treatment. This may be explained by the fact that these two processes likely recognize different pathophysiological mechanisms and, thus, different predictors. The achievement of a ≥20% reduction in PS (commonly adopted as the definition of “response”) is more likely to be associated with higher baseline PS volume needs and the absence of a residual endogenous GLP-2 secretion; on the contrary, the possibility of completely weaning off PS is more likely associated with lower baseline PS volume needs and the preservation of a certain amount of resorptive capacity by residual colon.

### 4.3. Efficacy by SBS Etiology

Our analysis did not show any significant difference in teduglutide efficacy according to SBS etiology. Nevertheless, a nonsignificant trend toward a slightly better outcome among patients with SBS due to Crohn’s disease could be observed, both in terms of response rate and weaning rate.

With respect to the response rate, a possible explanation for this finding might be related to the fact that PS-dependent patients due to Crohn’s disease are more likely to have no colon in continuity and higher PS volume needs [33,37,43], with the implications already described within the previous paragraph.

Regarding the weaning rate, the slight tendency towards an increased weaning probability among patients with Crohn’s disease held true even though the majority these patients did not have colon in continuity [33,37,43], which we showed to be a condition associated per se with lower weaning rates. Therefore, it might be argued that an SBS etiology due to Crohn’s disease might actually be a positive predictive factor for weaning, but that this could not be highlighted in our analysis due to the unavailability of individual patient data, and to the consequent impossibility of controlling the role of intestinal anatomy as a confounder and/or effect modifier. This reasoning, though speculative, would overall be in line with some preliminary findings by Joly et al. [43], which found that Crohn’s disease was not a predictor of weaning at univariate analysis (*p* = 0.91) but almost reached a statistical significance at multivariate regression after adjustment for age, intestinal length, and intestinal anatomy (*p* = 0.06).

### 4.4. Strengths and Limitations

This was the first meta-analysis that quantitatively assessed the efficacy of teduglutide in patients with SBS, providing pooled estimates of the response rate and the weaning rate among teduglutide-treated patients over time. It confirms and reinforces the findings of previous studies about the role of intestinal anatomy and SBS etiology as predictors of response. Moreover, it better clarifies their role as predictors of weaning, as no clear evidence was available in this regard.

Our analysis had some limitations. First, the robustness of the conclusions was limited by the low number of available studies and patients; this was particularly true for the subanalyses that examined the role of intestinal anatomy and SBS etiology as predictors of the outcome, whose results were weakened by the paucity of available data. Second, the quality was limited by that of the included studies, which were mostly characterized by an observational design; however, the risk of bias was generally moderate-to-low, which confirmed the likely small impact of this issue on the final results. Third, patients’ inclusion criteria and PS adjustment protocols were partly different between studies, and this could be responsible for a certain degree of heterogeneity in the considered outcomes; however, heterogeneity is a common limitation of all meta-analyses, and appropriate statistical methods—such as the use of a random-effect model—were adopted to account for it. Fourth, the comparison of response and weaning rates according to intestinal anatomy and SBS etiology were based on crude rate differences, as derived by univariate analyses; thus, the possible interplay between these predictors could not be evaluated.

## 5. Conclusions

In conclusion, our meta-analysis confirms the efficacy of teduglutide for PS reduction and/or discontinuation in adult patients with SBS, with a benefit increasing over time up to at least 1 year from treatment initiation. The presence of colon in continuity was a negative predictive factor for response but a positive predictive factor for weaning. An SBS etiology due to Crohn’s disease might be a positive predictive factor for both response and weaning, but the available data were insufficient to draw definite conclusions.

## Figures and Tables

**Table 1:** Study characteristics. Abbreviations: *N*, number; Prosp, prospective; PS, parenteral support; RCT, randomized controlled trial; Retr, retrospective; SBS, short bowel syndrome.

First Author, Year	Study Design	*N* of Patients	Age (Years) ^a^	Gender (% Male)	SBS Etiology (Crohn/Vascular/Other) (%)	Patients with Colon in Continuity (%)	Remnant Small Bowel Length (cm) ^a^	Time Since Start of PS Dependence (Years) ^a^	PS Volume at Baseline (L/Week) ^a^
Jeppesen et al., 2011 [27]	RCT	67 ^b^	48.6	44.8	34.4/32.8/32.8	67.2	53	6.9	11.1
		16 ^c^	49.4	43.8	43.7/18.8/37.5	68.8	53	7.9	10.7
Jeppesen et al., 2012 [28]	RCT	43 ^b^	50.9	48.8	23.3/30.2/46.5	60.5	138	6.8	12.9
		43 ^c^	49.7	44.2	18.6/37.2/37.2	53.5	123	5.9	13.2
Joly et al., 2019 [43]	Retr. cohort	54	52.3	64.8	29.6/38.9/31.5	64.8	62	9.8	11.2
Martin et al., 2020 [44]	Retr. cohort	31	51.0	64.5	32.3/32.3/35.4	51.6	74	4.8	7.5
O’Keefe et al., 2013 [45]	Prosp. cohort	52	48.1	53.8	34.6/34.6/30.8	71.2	60	7.0	11.5
Pevny et al., 2018 [46]	Retr. cohort	27	51.0	48.1	14.8/44.4/40.8	77.8	74	4.3	13.7
Puello et al., 2020 [47]	Retr. cohort	18	54.4	44.4	55.6/16.7/27.7	50.0	100	3.3	9.9
Schoeler et al., 2018 [48]	Prosp. cohort	14	50.5	35.7	50.0/35.7/14.3	64.3	50	5.2	12.2
Schwartz et al., 2016 [49]	Prosp. cohort	88	50.9	46.6	18.2/33.0/48.8	61.4	50	6.4	12.2
Solar et al., 2020 [50]	Prosp. cohort	17	40.2	52.9	0.0/47.1/52.9	94.1	38	6.5	12.1

^a^ Data reported as mean or median (according to the information provided by each study), ^b^ Treatment group, ^c^ Control group.

**Table 2:** Availability of efficacy data in terms of response rate, at different time points, for each included study; for each time point, the number of patients available for evaluation is reported in brackets.

First Author, Year	Available Time Points for the Evaluation of Teduglutide Efficacy in Terms of Response Rate		
	6 Months	1 Year	≥2 Years
Jeppesen et al., 2011 [27]	Yes ^a^ (56 pt.) ^b^	---	---
Jeppesen et al., 2012 [28]	Yes ^a^ (39 pt.) ^b^	---	---
Joly et al., 2019 [43]	Yes ^a^ (54 pt.)	---	---
Martin et al., 2020 [44]	---	Yes (31 pt.)	---
O’Keefe et al., 2013 [45]	---	Yes (43 pt.)	---
Pevny et al., 2018 [46]	Yes ^a^ (17 pt.)	Yes (11 pt.)	Yes (7 pt.)
Puello et al., 2020 [47]	--- ^c^	Yes (16 pt.)	Yes ^d^ (13 pt.)
Schoeler et al., 2018 [48]	Yes ^a^ (12 pt.)	Yes ^e^ (11 pt.)	---
Schwartz et al., 2016 [49]	---	---	Yes (65 pt.) ^f^
Solar et al., 2020 [50]	Yes ^a^ (17 pt.)	---	---

^a^ 24 weeks, ^b^ Number of patients in the treatment group, ^c^ Data of response rate at 6 months were reported in the original article, but have been excluded from our analysis due to inconsistency between the proportion of responders reported in terms of absolute numbers (16/18 patients) and the same data reported as a percentage (75%); this time point, however, was still included in the analysis concerning the weaning rate, as reported data were consistent in terms of this outcome measure, ^d^ Data at 2 years, 3 years, and 5 years were provided; in order to include the largest possible number of patients, data at 2 years were considered for quantitative analyses, ^e^ 48 weeks, ^f^ 35 patients at 24 months, 30 patients at 30 months. Abbreviations: pt, patients; ---, no available data.

## Data Availability

Not applicable.

## Supplementary Materials

The following supporting information can be downloaded at: https://www.mdpi.com/article/10.3390/nu14040796/s1. Table S1: risk of bias assessment of the RCTs according to RoB 2 tool; Table S2: risk of bias assessment of the observational studies according to ROBINS-I tool; Figure S1: Forest plot for the evaluation of the difference in response rate between the teduglutide and the placebo group in the available RCTs; Figure S2: subgroup analysis for the comparison of the response rate at 6 months in observational studies and RCTs; Figure S3: forest plot for the evaluation of the difference in response rate between the teduglutide and the placebo group in the available RCTs, after the exclusion of the patients treated with a teduglutide dosing schedule of 0.1 mg/kg/day; Figure S4: forest plot for the estimation of response rate at 6 months, 1 year, and ≥2 years, after the exclusion of the patients treated with a teduglutide dosing schedule of 0.1 mg/kg/day; Figure S5: subgroup analysis for the comparison of the response rate at 6 months in observational studies and RCTs, after the exclusion of the patients treated with a teduglutide dosing schedule of 0.1 mg/kg/day; Figure S6: Forest plot for the estimation of the weaning rate at 6 months, 1 year, and ≥2 years, after the exclusion of the patients treated with a teduglutide dosing schedule of 0.1 mg/kg/day.

## Appendix A

Electronic search strategy.

PUBMED
No filters applied
#1 Teduglutide
#2 #1 AND “short bowel syndrome”
#3 #1 AND “short gut syndrome”
#4 #1 AND “intestinal failure”
#5 #1 AND “intestinal insufficiency”
#6 #1 AND “intestinal deficiency”
#7 #1 AND “intestinal growth”
#8 #1 AND “intestinal adaptation”
#9 #1 AND “parenteral nutrition”
# 10 #1 AND “parenteral support”
#11 #1 AND “complications”
#12 #1 AND “cancer”
#13 #1 AND “dysplasia”

EMBASE
No filters applied
#1 Teduglutide
#2 #1 AND “short bowel syndrome”
#3 #1 AND “short gut syndrome”
#4 #1 AND “intestinal failure”
#5 #1 AND “intestinal insufficiency”
#6 #1 AND “intestinal deficiency”
#7 #1 AND “intestinal growth”
#8 #1 AND “intestinal adaptation”
#9 #1 AND “parenteral nutrition”
# 10 #1 AND “parenteral support”
#11 #1 AND “complications”
#12 #1 AND “cancer”
#13 #1 AND “dysplasia”

COCHRANE LIBRARY
No filters applied
#1 Teduglutide
#2 #1 AND “short bowel syndrome”
#3 #1 AND “short gut syndrome”
#4 #1 AND “intestinal failure”
#5 #1 AND “intestinal insufficiency”
#6 #1 AND “intestinal deficiency”
#7 #1 AND “intestinal growth”
#8 #1 AND “intestinal adaptation”
#9 #1 AND “parenteral nutrition”
# 10 #1 AND “parenteral support”
#11 #1 AND “complications”
#12 #1 AND “cancer”
#13 #1 AND “dysplasia”

OVID
No filters applied
#1 Teduglutide
#2 #1 AND “short bowel syndrome”
#3 #1 AND “short gut syndrome”
#4 #1 AND “intestinal failure”
#5 #1 AND “intestinal insufficiency”
#6 #1 AND “intestinal deficiency”
#7 #1 AND “intestinal growth”
#8 #1 AND “intestinal adaptation”
#9 #1 AND “parenteral nutrition”
# 10 #1 AND “parenteral support”
#11 #1 AND “complications”
#12 #1 AND “cancer”
#13 #1 AND “dysplasia”

CINAHL
No filters applied
#1 Teduglutide
#2 #1 AND “short bowel syndrome”
#3 #1 AND “short gut syndrome”
#4 #1 AND “intestinal failure”
#5 #1 AND “intestinal insufficiency”
#6 #1 AND “intestinal deficiency”
#7 #1 AND “intestinal growth”
#8 #1 AND “intestinal adaptation”
#9 #1 AND “parenteral nutrition”
# 10 #1 AND “parenteral support”
#11 #1 AND “complications”
#12 #1 AND “cancer”
#13 #1 AND “dysplasia”
