# Supplementary material for: Efficacy of Teduglutide for Parenteral Support Reduction in Patients with Short Bowel Syndrome: A Systematic Review and Meta-Analysis
Source: Nutrients. 2022 Feb 14;14(4):796. doi: 10.3390/nu14040796 (PMC8880479; doi:10.3390/nu14040796)
Supplement: Supplementary file 1 [file nutrients-14-00796-s001.zip › nutrients-1579314-supplementary.pdf]

Supplementary tables and figures

Table S1. Risk of bias assessment of the RCTs according to RoB 2 tool.

**Study**	**Bias arising from the randomisation process**	**Bias due to deviations from intended interventions**	**Bias due to missing outcome data**	**Bias in measurement of the outcome**	**Bias in selection of the reported result**	**Overall risk of bias**
Jeppesen et al, 2011 [27]	Low	Low	Low	Low	Low	Low
Jeppesen et al, 2012 [28]	Low	Low	Low	Low	Low	Low

Abbreviations: RCT, randomized controlled trial.

Table S2. Risk of bias assessment of the observational studies according to ROBINS-I tool.

**Study**	**Bias due to confounding**	**Bias in selection of participants into the study**	**Bias in measurement classification of interventions**	**Bias due to deviations from intended interventions**	**Bias due to missing data**	**Bias in measurement of outcomes**	**Bias in selection of the reported result**	**Overall bias**
Joly et al, 2019 [41]	Low	Low	Low	Low	Low	Low	Low	Low
Martin et al, 2020 [42]	Moderate	Moderate	Low	Low	Low	Low	Low	Moderate
O'Keefe et al, 2013 [43]	Low	Low	Low	Low	Low	Low	Low	Low
Pevny et al, 2018 [44]	Low	Low	Low	Low	Low	Low	Low	Low
Puello et al, 2020 [45]	Low	Low	Low	Low	Moderate	Low	Low	Moderate
Schoeler et al, 2018 [46]	Moderate	Moderate	Moderate	Low	Low	Low	Low	Moderate
Schwartz et al, 2016 [47]	Moderate	Moderate	Low	Low	Low	Low	Low	Moderate
Solar et al, 2020 [48]	Moderate	Moderate	Low	Low	Low	Low	Low	Moderate

Figure S1. Forest plot for the evaluation of the difference in response rate between the teduglutide and the placebo group in the available RCTs.

0.

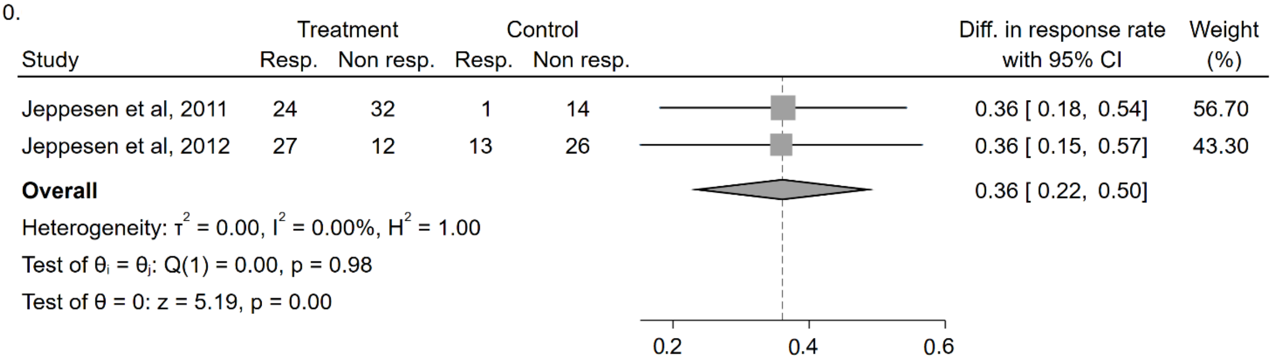

Abbreviations: CI, confidence interval; Diff., difference; RCT, randomized controlled trial; Resp., responders; Non resp., non responders.

.

Figure S2. Subgroup analysis for the comparison of the response rate at 6 months in observational studies and RCTs.

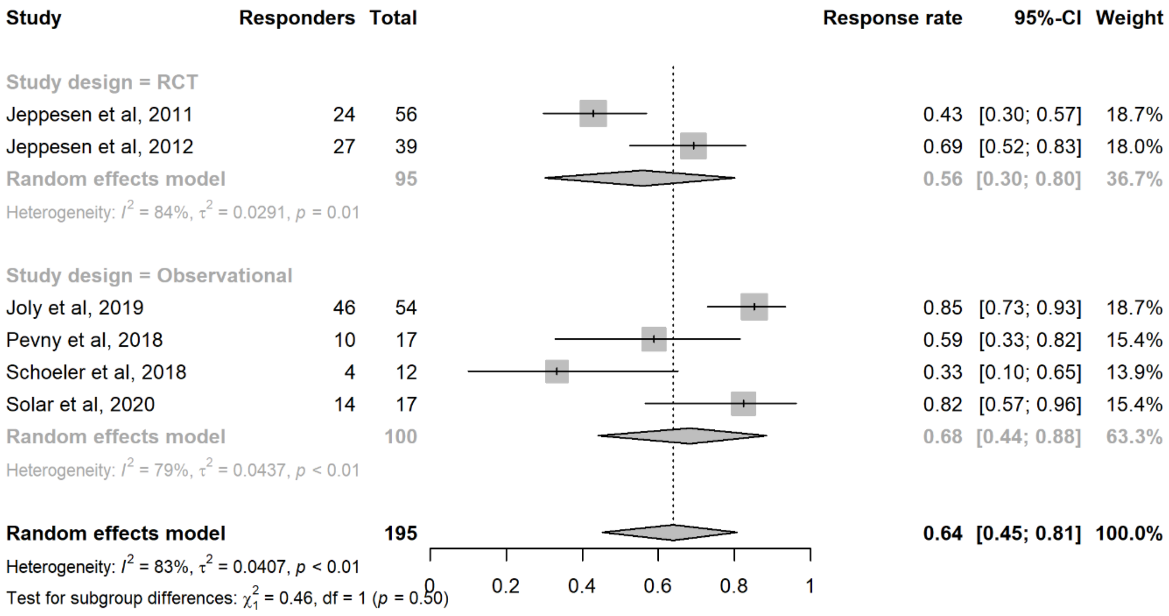

Abbreviations: CI, confidence interval; RCT, randomized controlled trial.

Figure S3. Forest plot for the evaluation of the difference in response rate between the teduglutide and the placebo group in the available RCTs, after the exclusion of the patients treated with a teduglutide dosing schedule of 0.1 mg/kg/day.

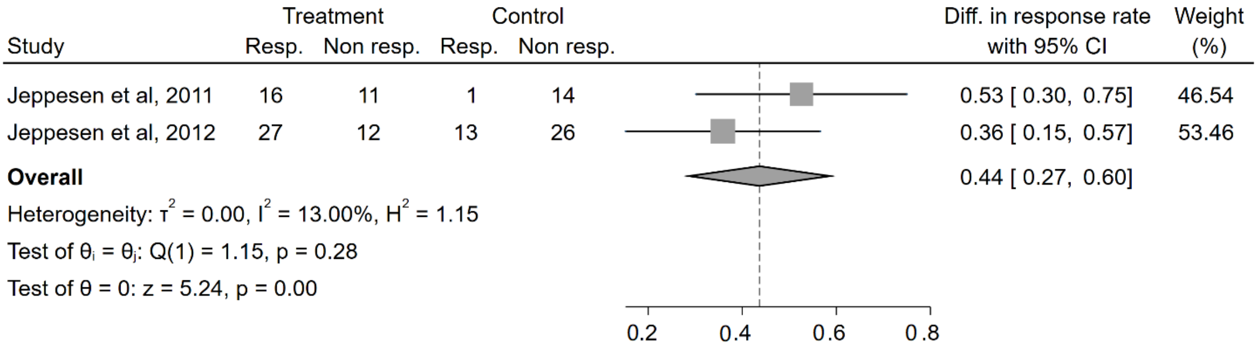

Abbreviations: CI, confidence interval; Diff., difference; RCT, randomized controlled trial; Resp., responders; Non resp., non responders.

Figure S4. Forest plot for the estimation of response rate at 6 months, 1 year, and ≥ 2 years, after the exclusion of the patients treated with a teduglutide dosing schedule of 0.1 mg/kg/day.

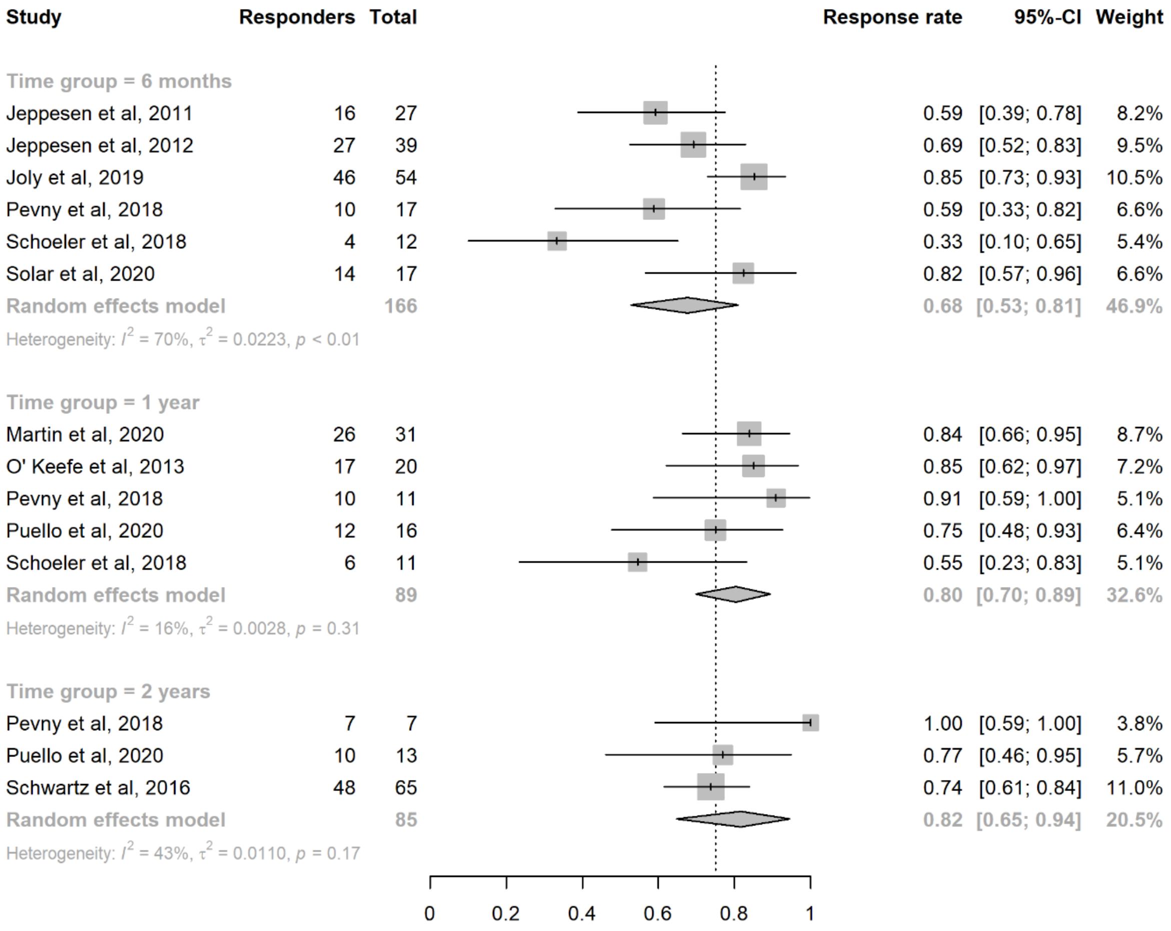

Abbreviations: CI, confidence interval.

Figure S5. Subgroup analysis for the comparison of the response rate at 6 months in observational studies and RCTs, after the exclusion of the patients treated with a teduglutide dosing schedule of 0.1 mg/kg/day.

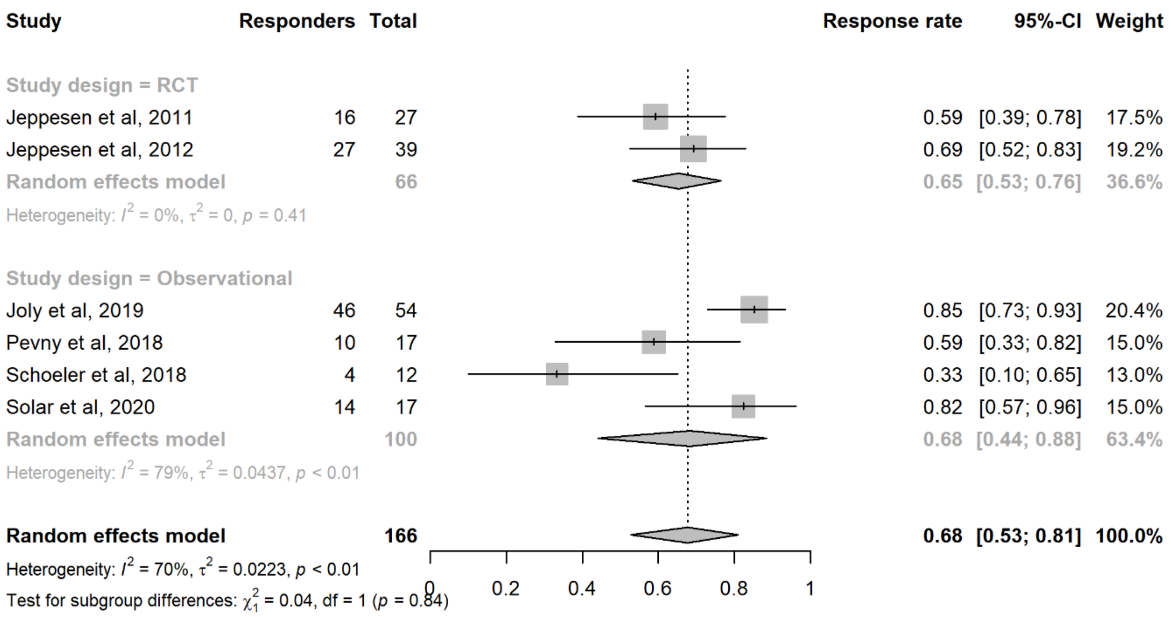

Abbreviations: CI, confidence interval; RCT, randomized controlled trial.

Figure S6. Forest plot for the estimation of the weaning rate at 6 months, 1 year, and ≥ 2 years, after the exclusion of the patients treated with a teduglutide dosing schedule of 0.1 mg/kg/day.

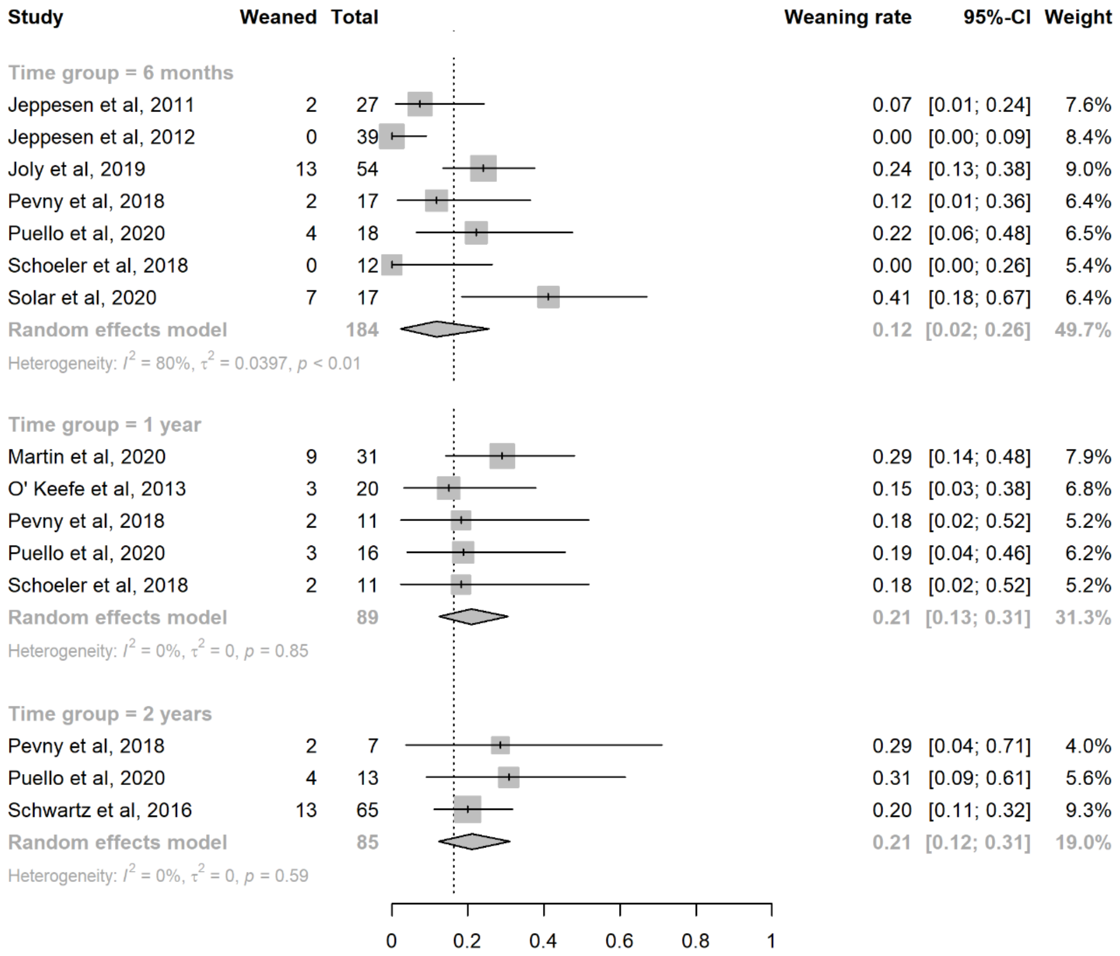

Abbreviations: CI, confidence interval.
